# Inhibition of class I HDACs preserves hair follicle inductivity in postnatal dermal cells

**DOI:** 10.1038/s41598-021-03508-0

**Published:** 2021-12-15

**Authors:** Minji Park, Sunhyae Jang, Jin Ho Chung, Ohsang Kwon, Seong Jin Jo

**Affiliations:** 1 Department of Dermatology, Seoul National University College of Medicine, Seoul, Republic of Korea; 2 Laboratory of Cutaneous Aging and Hair Research, Biomedical Research Institute, Seoul National University Hospital, Seoul, Republic of Korea; 3 Institute of Human-Environment Interface Biology, Medical Research Center, Seoul National University, Seoul, Republic of Korea; 4 Department of Biomedical Sciences, Seoul National University College of Medicine, Seoul, Republic of Korea

**Keywords:** Cell biology, Stem cells

## Abstract

Induction of new hair follicles (HFs) may be an ultimate treatment goal for alopecia; however, functional cells with HF inductivity must be expanded in bulk for clinical use. In vitro culture conditions are completely different from the in vivo microenvironment. Although fetal and postnatal dermal cells (DCs) have the potential to induce HFs, they rapidly lose this HF inductivity during culture, accompanied by a drastic change in gene expression. This suggests that epigenetic regulation may be involved. Of the various histone deacetylases (HDACs), Class I HDACs are noteworthy because they are ubiquitously expressed and have the strongest deacetylase activity. This study revealed that DCs from postnatal mice rapidly lose HF inductivity and that this reduction is accompanied by a significant decrease in histone H3 acetylation. However, MS-275, an inhibitor of class I HDACs, preserves HF inductivity in DCs during culture, increasing alkaline phosphatase activity and upregulating HF inductive genes such as BMP4, HEY1, and WIF1. In addition, the inhibition of class I HDACs activates the Wnt signaling pathway, the most well-described molecular pathway in HF development, via increased histone H3 acetylation within the promoter region of the Wnt transcription factor LEF1. Our results suggest that class I HDACs could be a potential target for the neogenesis of HFs.

## Introduction

The survival, proliferation, and differentiation of cells are determined by both their intrinsic potential and the signal cues provided by the microenvironment in which they reside^1^. In vitro expansion of adult stem cells is a powerful tool for tissue engineering but remains technically challenging as removing adult stem cells from their native niche can make it hard to sustain their native differentiation and proliferation capabilities^2^.

Induction of new hair follicles (HFs) may be an ultimate treatment goal for alopecia; however, cells with HF inductivity must be expanded in bulk for clinical use. Fetal and neonatal mice dermal cells (DCs) still have the ability to differentiate dermal condensate of HFs^3,4^, which they lose in adults. This means that when they are mixed with epidermal cells and injected subcutaneously, they produce new HFs in about 2 weeks. However, DCs lose their potency when cultured ex vivo, especially when these conditions are significantly different from their in vivo microenvironment. A prime example of this is dermal papilla cells that hardly ever induce HFs after in vitro culture expansion^5,6^. Cultured cells present with a significant alteration in their gene expression patterns, suggesting that these functional differences may be associated with changes in their epigenetic regulation^5–7^.

Histone modification plays a fundamental role in most biological processes associated with the manipulation or expression of the DNA. The modifications not only change the chromatin structure but can also respond to external signals to regulate gene transcription^7,8^. Histone deacetylases (HDACs) are representative histone-modifying enzymes that can control gene transcription via histone tail acetylation^9^ and have thus been targeted in many regeneration studies^10,11^. There are several classes of HDACs; the most common being Class I, which includes HDAC1, HDAC2, HDAC3, and HDAC8. These HDACs are ubiquitously expressed and regulate a variety of cellular events^12^. Class I HDACs exhibit different affinity for and activity against acetylated substrates when compared with other classes of HDACs^13^, and while most class I HDACs possess potent deacetylase activity, class II HDACs are enzymatically inactive when on their own and have to be accompanied by Class I HDAC complexes^13^. MS275 (Entinostat), a synthetic benzamide derivative, inhibits typical Class I HDACs by blocking their deacetylation of the zinc ion (Zn^2+^) in the catalytic pocket^14^. This compound not only displays anti-proliferative activity in several cancer cell lines^15,16^, but also has been shown to have several potential roles in tissue engineering^17,18^.

In this study, we examined the expression of HF inductive genes and the state of histone acetylation in cultured DCs from postnatal mice and screened selective HDAC inhibitors to identify an HF inductivity protecting effect. We found that inhibition of class I HDACs preserved the HF inductivity of postnatal DCs during culture via the activation of the Wnt signaling pathway and histone H3 acetylation in the promoter region of LEF1.

## Results

### DCs from postnatal mice rapidly lose HF inductivity during in vitro culture, which is accompanied by a decrease in histone H3 acetylation

**Figure 1:**
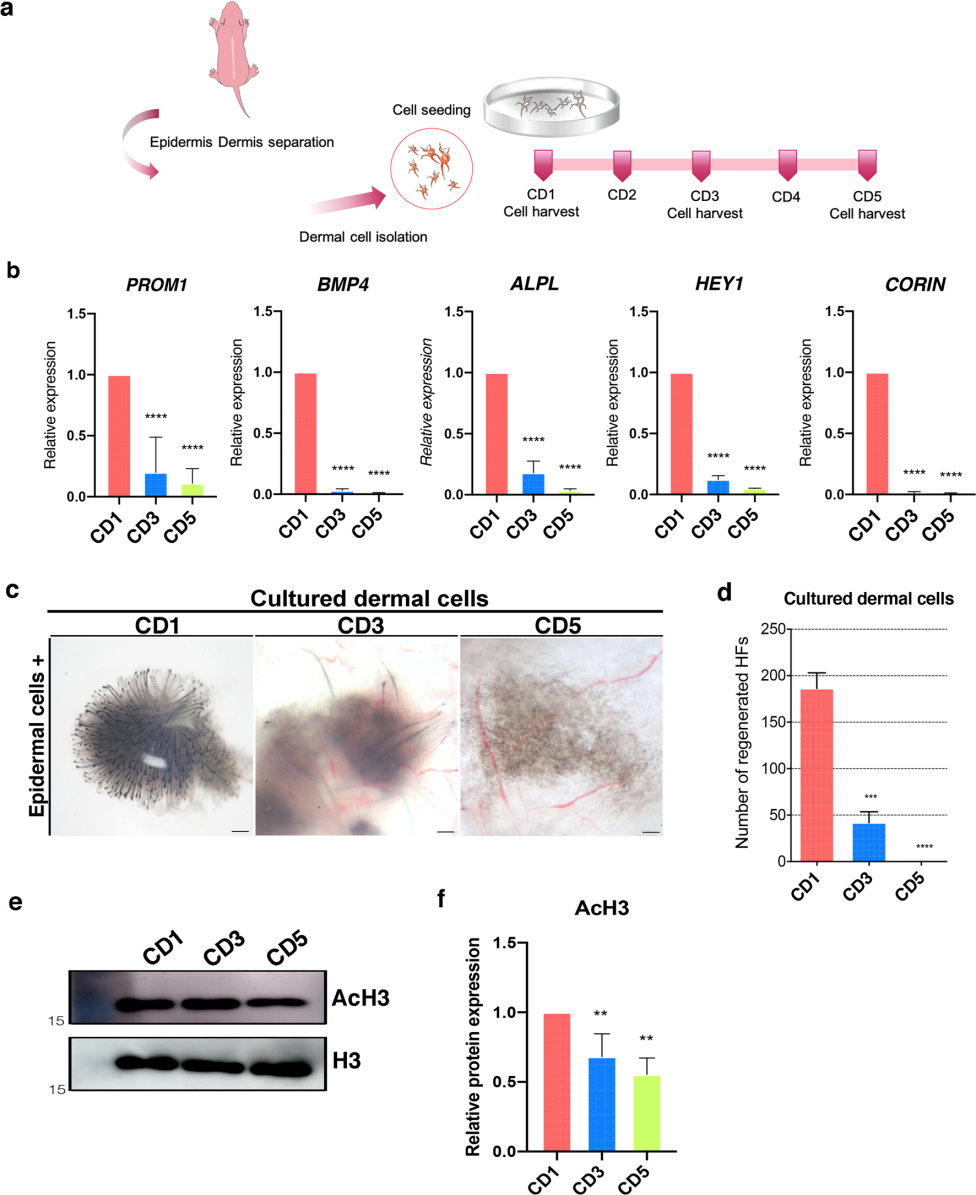
HF inductivity and histone H3 acetylation in cultured DCs from postnatal mice. (**a**) Schematic describing DC preparation and culture. (**b**) Relative expression of signature genes for HF inductivity in cultured DCs at CD1, CD3, and CD5. Data are shown as the mean ± S.E.M. (*, *P* < 0.05; **, *P* < 0.01; ***, *P* < 0.001; ****, *P* < 0.0001) (n = 6). (**c**) HF reconstitution assay using CD1, CD3, and CD5 DCs and freshly isolated epidermal cells (scale bar 500 μm), and (**d**) the number of reconstituted HFs as determined by ImageJ (n = 5). (**e**) Immunoblots against acetylated histone H3 and total H3 for each day of culture (n = 5). (**f**) Relative histone H3 acetylation expression graph shown in (**e**). Data are represented as the mean ± S.E.M. (n = 4) (**, *P* < 0.01). CD, culture day; DCs, dermal cells; HFs, hair follicles.

We first determined the duration for which DCs from neonatal mice retain their HF inductivity during in vitro culture*.* To this end, we cultured freshly isolated neonatal dermal cells for up to 5 days and then harvested them on culture day 1 (CD1), 3 (CD3), and 5 (CD5) (Fig. 1a). We then examined the mRNA levels of several signature genes for neonatal dermal papilla and dermal condensate^19,20^ in each sample. This evaluation revealed that the expression of the signature genes for HF inductivity, CORIN, BMP4, ALPL, HEY1, and PROM1, all progressively decreased from CD1 to CD5 (Fig. 1b). Next we confirmed the loss of HF inductivity by completing an HF reconstitution assay or so-called patch assay^3^, using freshly isolated epidermal cells from C57/BL6 mice and DCs from CD1, 3, and 5. This assay revealed that at the 2 week mark, the CD1 DCs produced several HFs, whereas the CD3 DC assay produced significantly fewer HFs under the same conditions; the CD5 samples produced no HFs at all (Fig. 1c, d). These findings suggest that murine DCs quickly lose their HF inductivity during in vitro culture.

Histone H3 acetylation is closely associated with the regulation of gene expression during mesenchymal cell fate determination and differentiation^21,22^, and has been reported to regulate BMPs and other genes related to HF inductivity^23^. Given this, we went on to examine the level of histone H3 acetylation during in vitro DC culture. We extracted histones from CD1, CD3, and CD5 DCs and confirmed that their histone H3 acetylation decreased in a time-dependent manner using western blot (Fig. 1e, f).

### DCs treated with MS275 retain HF inductivity during culture and successfully reconstitute HFs

**Figure 2:**
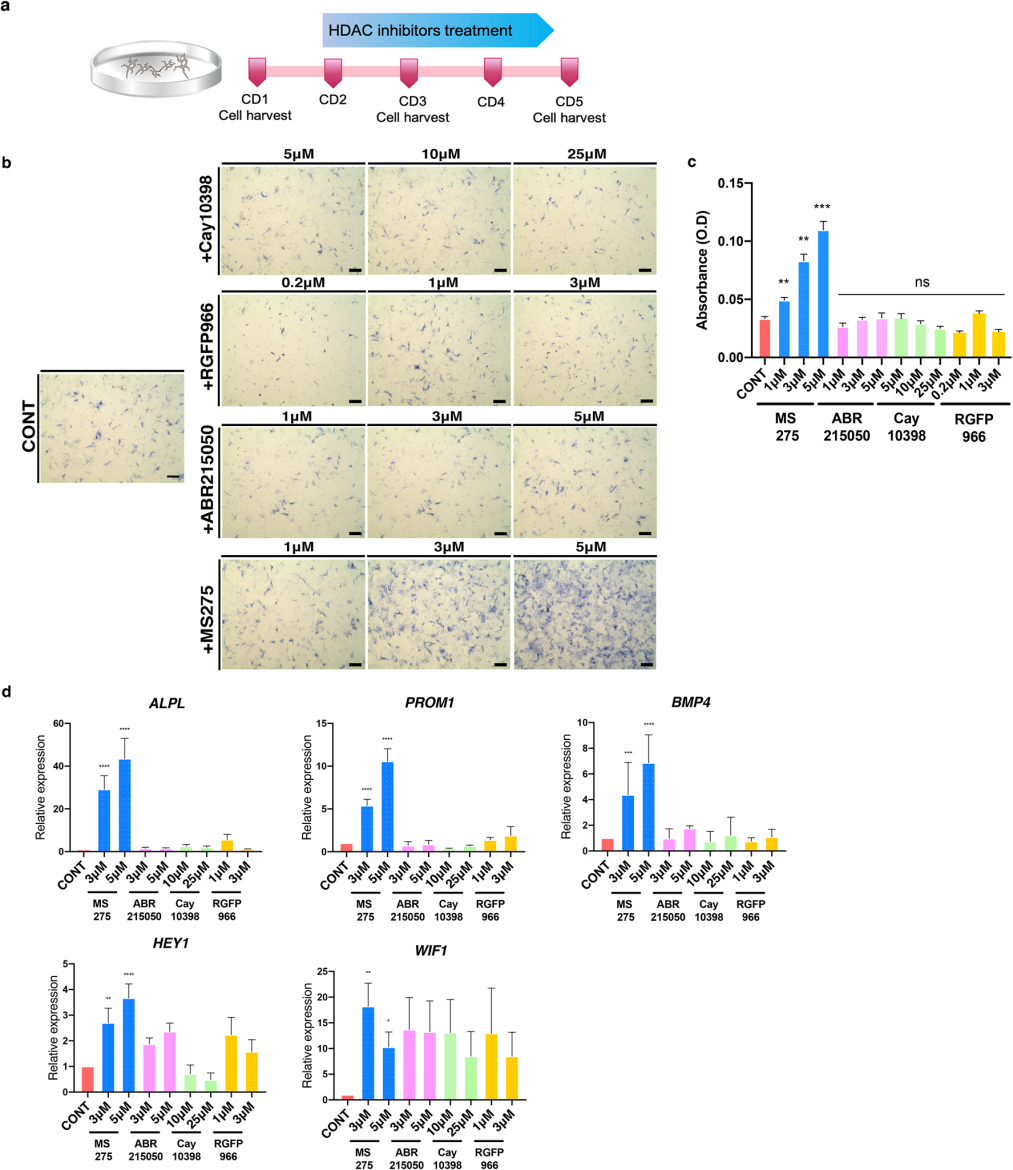
Effect of HDAC inhibition on cultured DCs from postnatal mice. (**a**) Schematic describing the in vitro culture of DCs, highlighting their treatment with several HDAC inhibitors starting from CD2. (**b**) ALP activity of the DCs cultured with CAY10398, RGFP966, ABR215050, and MS275. Scale bar, 200 μm. (**c**) Bar graphs represent the mean of optical density(595 nm) by three replicated experiments. Data are represented man ± S.E.M (n = 3) (ns, *P* ≥ 0.05; **, *P* < 0.01; ***, *P* < 0.001). (**d**) Relative expression of the signature genes for HF inductivity in DCs treated with various HDAC inhibitors. Data are shown mean ± S.E.M. Each experiment replicate (n = 3) and analyzed by t-test (*, *P* < 0.05; **, *P* < 0.01; ***, *P* < 0.001; ****, *P* < 0.0001). ALP, alkaline phosphatase; CD, culture day; CONT, controls; DCs, dermal cells; HDAC, histone deacetylase; HF, hair follicle.

**Figure 3:**
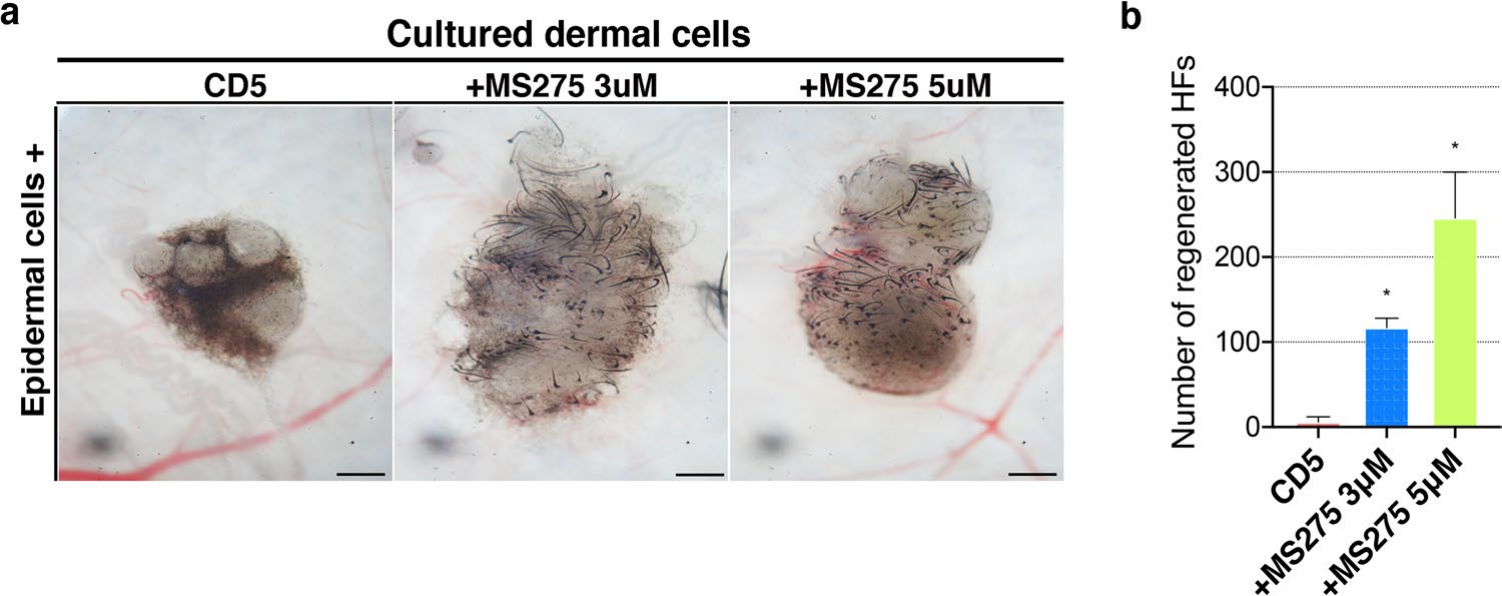
HF reconstitution with the DCs treated with MS275, an inhibitor of class I HDACs. (**a**) Successful reconstitution of HFs using MS275 treated DCs and fresh epidermal cells at a ratio of 2:1. Scale bar, 200 μm. (**b**) The number of reconstituted HFs as determined by Image J software. (*, *P* < 0.05). CD, culture day; DCs, dermal cells; HFs, hair follicles.

We then wanted to establish whether the loss in HF inductivity in these cultured DCs was related to this decrease in histone H3 acetylation and if it could be prevented by the inhibition of histone deacetylation. To date 17 putative HDACs have been identified and classified into three distinct families^24^; accordingly various selective HDAC inhibitors have been developed for clinical applications^25^. Here, we examined several selective HDAC inhibitors including CAY10398 (an HDAC1 inhibitor)^26^, RGFP966 (an HDAC3 inhibitor)^27^, ABR215050 (an HDAC4 inhibitor)^28^, and MS275 (a class I HDAC inhibitor)^29^ in cultured DCs over four consecutive days (Fig. 2a). Treatment with these compounds induced no prominent changes in cell morphology, but cell proliferation was markedly decreased when treated with 25 µM CAY10398 (data not shown). It is well known that alkaline phosphatase (ALP) activity is a useful marker for identifying stemness in cells. Its expression is increased in osteogenic, embryonic, and kidney stem cells^30^ as well as in dermal papilla cells. Therefore, it is often used to detect mesenchyme-derived cells^31^. Given this, we went on to measure the ALP activity in our cells after treatment with the various HDAC inhibitors and found that MS275 significantly increased ALP activity while the other selective inhibitors had no effect on this marker (Fig. 2b, c). Likewise, the mRNA expression levels of the signature genes for hair inductivity such as BMP4, HEY1, and WIF1 were markedly increased in MS275 treated DCs (Fig. 2d), and the HF reconstitution assay showed that DCs cultured with MS275 were able to successfully reconstitute HFs even after 5 days of culture (Fig. 3a). In addition, the number of HFs was significantly increased with increasing concentrations of MS275 (Fig. 3b). Taken together this data suggests that the collective inhibition of class I HDACs, rather than the targeted inhibition of a single specific HDAC, effectively preserves HF inductivity in cultured DCs.

### MS275 activates the Wnt signaling pathway

We then went on to evaluate the effects of MS275 treatment on Wnt signaling in these DCs as several studies have demonstrated a correlation between Wnt signaling and HF inductivity^32,33^.

**Figure 4:**
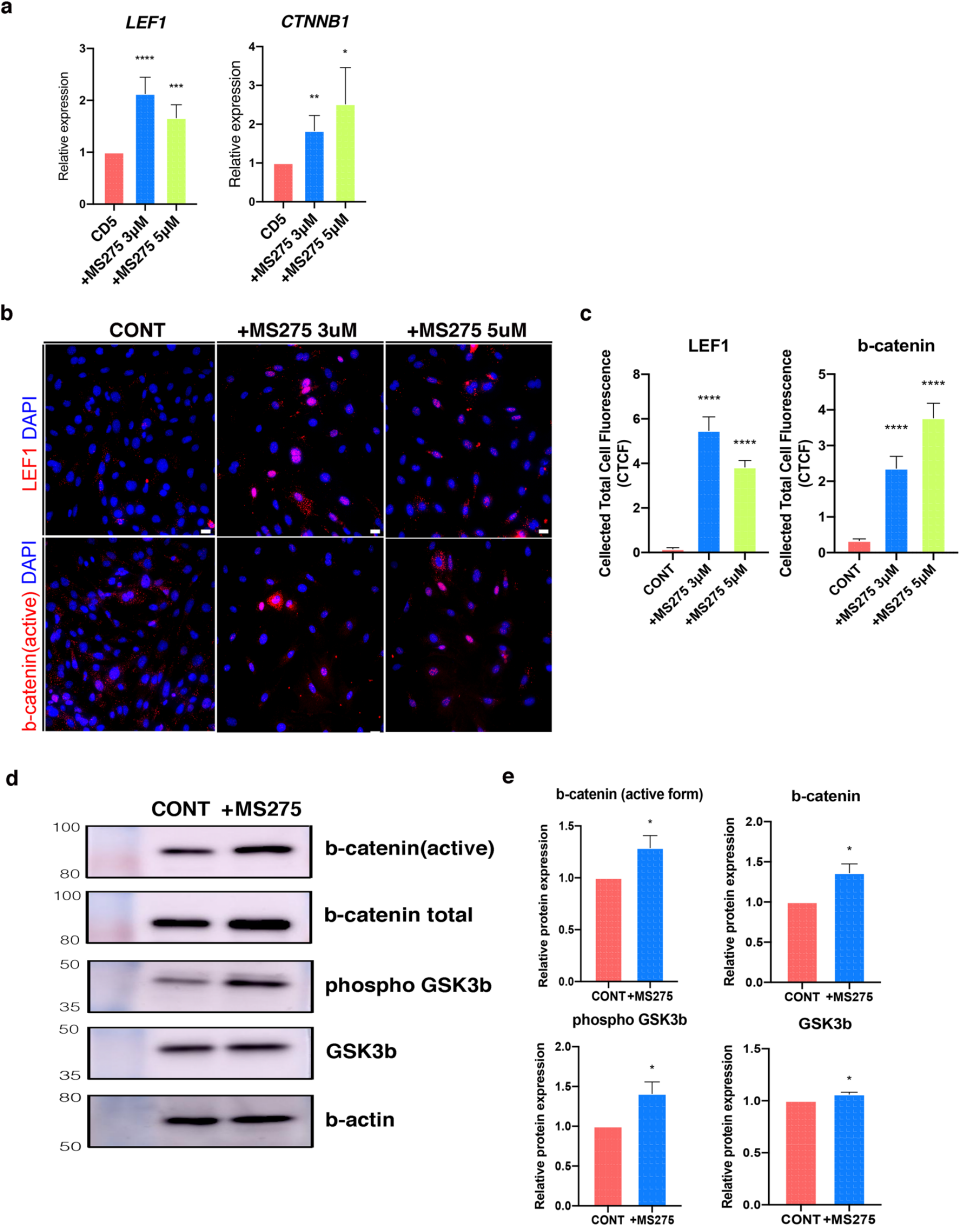
Activated Wnt signaling pathway by the inhibition of class I HDACs. (**a**) Gene expression of Wnt transcription factor LEF1 and CTNNB1 in MS275 treated DCs. Data are shown as the mean ± S.E.M and evaluated by t-test (*, *P* < 0.05; **, *P* < 0.01; ***, *P* < 0.001; ****, *P* < 0.0001). (**b**) Immunostaining against LEF1 and active β-catenin in MS275 treated DCs. Scale bar, 10 μm. (**c**) Corrected Total Cell Fluorescence (CTCF) graphs of CONT and MS275 treated DCs. The fluorescent intensity from the confocal image of each DCs was determined by ImageJ software. Data were shown as means ± S.E.M in each group. (n = 50) (****, *P* < 0.0001 vs CONT). (**d**) Immunoblots against β-catenin and GSK-3β from the Wnt signaling pathway in the total protein of MS275 treated DCs. (**e**) The protein expression level shown in (d) were quantified. Data were represented as the mean ± S.E.M. (n = 3) (*, *P* < 0.05). CONT, controls; DCs, dermal cells.

Our evaluations revealed that several Wnt signaling pathway-related genes, including LEF1, CTNNB1, AXIN2, WNT5A, and TCF6, were significantly upregulated in MS275 treated CD5 DCs (Figs. 4a and SFigure 1).

Immunofluorescence analysis also showed that both LEF1 and β-catenin were significantly increased in MS275 treated DCs (Fig. 4b, c) and western blot analysis revealed that both the expression of total and activated β-catenin increased in MS275 treated CD5 DCs. GSK3β, which plays a role in β-catenin degradation^34^, is regulated through inhibitory serine-phosphorylation^35^. Our data shows that there was an increase in phosphorylated GSK3β in response to MS275 treatment which may lead to a reduction in β-catenin degradation (Fig. 4d, e). These findings suggested that the inhibition of class I HDACs was associated with the activation of DCs via the Wnt signaling pathway.

### Histone H3 acetylation is associated with the regulation of LEF1

**Figure 5:**
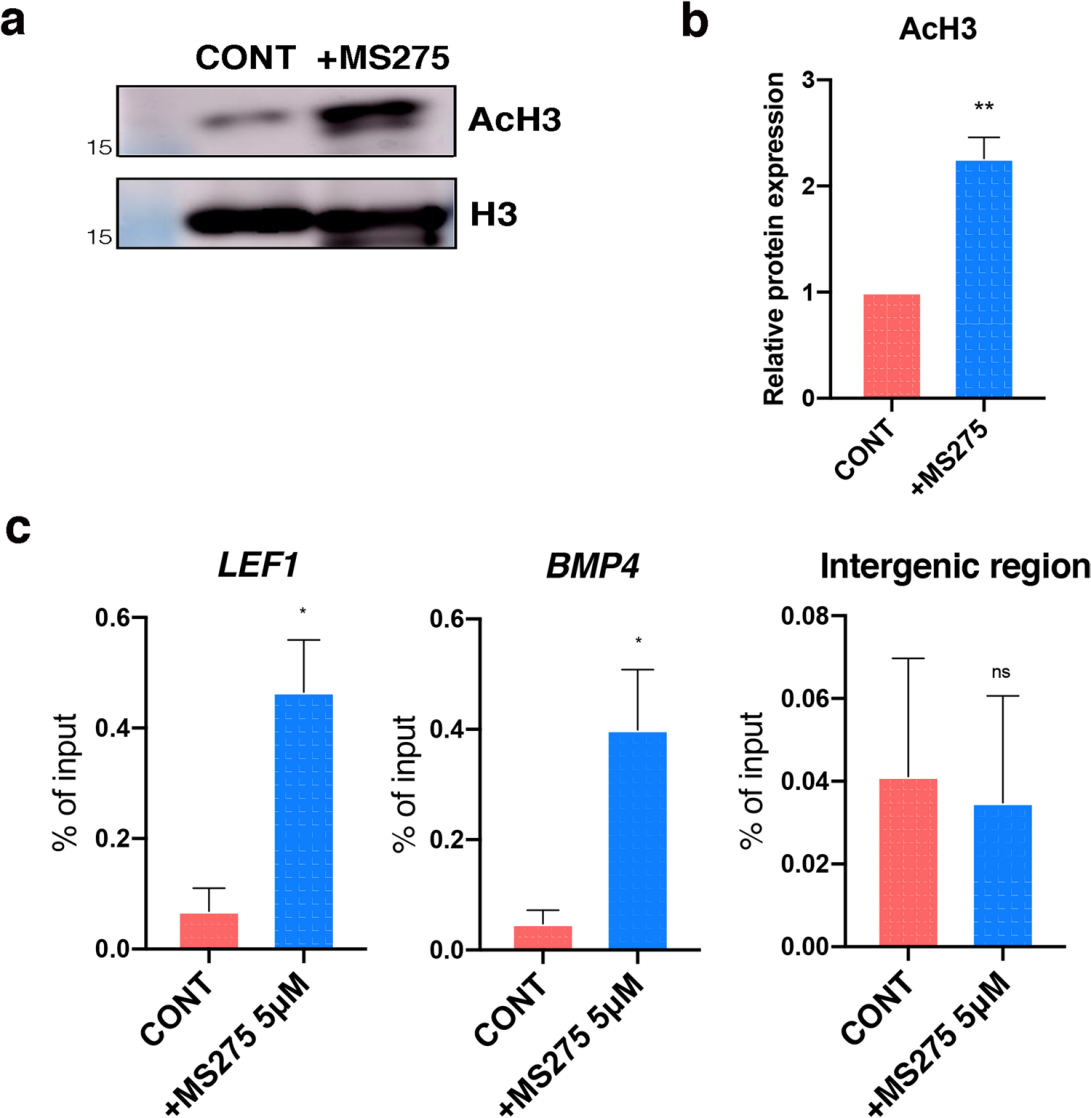
Histone H3 acetylation at the promoter regions of LEF1 and BMP4. (**a**) Histone H3 acetylation in MS275 treated DCs compared to CONT DCs. (**b**) The expression level of Histone H3 acetylation quantified by AMERSHAM imagequant software. Data were shown as the mean ± S.E.M (n = 3) (**, *P* < 0.01). (**c**) ChIP-qPCR using acetylated H3 antibody against the promoter regions of LEF1 and BMP4 in control and treated DCs. An intergenic region primer was used as a negative control and samples were normalized against the input DNA and represented as % of input. Data are shown as the mean ± S.E.M (n = 3) (*, *P* < 0.05). CONT, controls; DCs, dermal cells.

We also examined the association between histone H3 acetylation and the regulation of the Wnt signaling pathway. As expected, western blot showed that histone H3 acetylation increased in the MS275 treated CD5 DCs (Fig. 5a, b). In addition, the ChIP assay for LEF1, a transcription factor mediating the cell’s nuclear response to Wnt signaling, revealed that the level of H3 acetylation within the promoter region of the LEF1 gene was significantly increased in MS275 treated DCs (Fig. 5c), implicating H3 acetylation in the direct regulation of LEF1 expression. ChIP assay of the promoter region of BMP4, another HF inductive gene that upregulates LEF1 expression, showed a similar result.

## Discussion

During organogenesis, HF morphogenesis is initiated by signal from DCs to epidermal cells, and reciprocal interaction between dermal papilla precursors and overlying epithelial cells contribute to the development of the HF^36^. In recent decades, HF biology has witnessed an increase in the knowledge of key processes such as genesis and cycling; however, alopecia is difficult to manage because existing surgical and pharmacological treatments are not effective. Recently, HF bioengineering has been suggested as a novel treatment approach, which is based on the development of fully functional HFs with cycling activity from an expanded population of trichogenic cells^37^. Among them, stem cell based tissue engineering using iPSC^38,39^ or 3D organ culture dermal papillae^6,40^ is emerging as the most potential approach to reconstruct HF in vitro.

Although DCs are described as a heterogeneous population of cells mainly comprising fibroblasts^4,20^, fetal and postnatal populations of these cells retain some of their progenitor cells. These progenitor cells have the potential to differentiate into diverse cell types in the dermis, suggesting the fact that freshly isolated DCs from postnatal mice retain HF inductivity and can be used for HF neogenesis and bioengineering if they can be expanded in bulk in vitro. However, maintaining cell properties in vitro is difficult. Cells begin to change as soon as they are seeded onto culture plates, and they fully adapt to culture conditions within a few days of expansion and proliferation.

Along these lines, we established that the level of histone H3 acetylation in DCs was significantly reduced in a short period of time after culture, which was effectively recovered by MS275, an HDAC class I inhibitor. Epigenetic modification and these inhibitors are useful tools to maintain or recover cell characteristics^41,42^, and there are many histone modification sites to regulate gene expression in specific cells. In mesenchymal stem cells, the promoters associated with RUNX2 and ALP increased H3 acetylation level during differentiation^22^. Furthermore, studies have indicated that histone H3K9, 14 directly regulates the BMP2, BMP4 and BMP6 promoter region in mouse skin -derived precursors^23^.

In this study, we screened several inhibitors of class I HDACs (HDAC1, 2, 3, and 8) as they are ubiquitously expressed and have strong deacetylase activity when compared with other classes of HDACs^43^. We found that MS275, a strong inhibitor of HDAC1, 2, and 3, successfully upregulated the expression of HF-inductive genes in cultured DCs, whereas CAY10398 and RGFP966, highly selective inhibitors of HDAC1 and HDAC3, respectively, did not. We also examined ABR215050, a selective inhibitor of HDAC4 that belongs to class II HDACs. Although class II HDACs usually exhibit low enzymatic activity levels, HDAC4 with an H976Y (histidine to tyrosine) mutation demonstrates strong deacetylase activity^44^, suggesting the fact that HDAC4 may also be involved in the deacetylation of the histones in the HF-inductive genes. However, our results revealed that HDAC4 inhibition had little effect on HF signature genes (Fig. 2b) and Wnt signaling pathway activation (SFigure 2). Collectively, these results suggested that class I HDACs were principally and complementarily responsible for the loss of HF inductivity in DCs as opposed to the activity of a single, specific class I or class II HDAC. Further studies using various selective HDAC inhibitors may be necessary to elucidate this regulatory mechanism.

We established that histone H3 acetylation activates Wnt signaling in the cultured DCs. The Wnt signaling pathway is the most well characterized molecular pathway in HF morphogenesis, growth, and cycle^45,46^. During HF morphogenesis, epidermal Wnt signaling activates the DCs, and reciprocal downstream signaling leads to HF development^47,48^. Moreover, recent studies suggest that Wnt signaling maintains HF inductivity in dermal papilla cells during culture^32^, indicating that Wnt signaling is essential for HF development. Transcription factor LEF1 is not only an established target of Wnt but also a key player in activating Wnt signaling. It directly regulates the nuclear localization of β-catenin, an indicator of active Wnt signaling^49^, and acts as a partner to various other transcription factors and signaling pathways. The BMP signaling pathway is another critical pathway associated with the orchestration of tissue architecture, and this pathway also upregulates LEF1 expression as the locations of the LEF1, BMP2/4, and WNT3A genes are very close together and, in fact, one of the BMP regulatory elements overlaps with the LEF1 promoter^50^. We found that BMP4 expression is regulated by histone H3 acetylation in the cultured DCs using the ChIP assay. Taken together, our results indicate that histone acetylation orchestrates upstream of the Wnt and BMP signaling pathways. We plan to investigate the interactions between these two molecular pathways in DCs in our next study.

This study demonstrated that the inhibition of class I HDACs preserves HF inductivity in cultured DCs via Wnt signaling pathway activation in response to changes in H3 acetylation. Moreover, our study established that HDAC inhibitors may have several functional applications in developing effective xeno-free culture conditions^51^ and specifically showed that MS275 could be used as an effective epigenetic modifier to manipulate postnatal DCs in vitro*.* Future studies will help us understand how these interactions are regulated, and they must evaluate whether these treatments can also be applied in vivo.

## Methods

### Animal

All animal experimental procedure complied with the ARRIVE (Animal Research: Reporting in Vivo Experiments) guidelines2.0 for preclinical animal studies and were approved by the Institutional Animal Care and Use Committee of Seoul National University Hospital (SNUH-IACUC 19-0135). All experiments were performed in accordance with relevant guidelines and regulations. C57BL/6N and BALB/c-nu mice were obtained from ORIENTBIO Inc, Korea. All animals housed in the specific-pathogen-free laboratory animal room at Seoul National University Hospital.

### Mouse DC isolation and culture

Whole skin samples from postnatal C57BL/6 N mice were floated on 1 mg/mL DispaseII (ROCHE) for 30 min at 37 °C, in order to separate the epidermis and dermis. Separated dermis was then placed in a plate with LiberaseTL (ROCHE) for 15–40 min at 37 °C and isolated DCs were filtered through a 100 µM (SPL) to 40 µM (BD) cell strainer and centrifuged to remove any additional cell debris. Collected cells were cultured in DMEM (WELLGENE) supplemented with 10% FBS.

### Histone extraction and western blot

Histones were extracted using an EpiQuick total histone extraction kit (EPIGENTEK) according to the manufacturer’s instructions. Equal amounts of histones were separated on 15% acrylamide gels and electrophoretically transferred to PVDF membranes. These membranes were then incubated with primary antibodies against acetylated histone H3 (MILLIPORE) and histone H3 (INVITROGEN) at 4 °C overnight. The next day, the membranes were washed and then incubated with the appropriate HRP- conjugated secondary IgG antibody (GENETEX) at 25 °C for 1 h. These complexes were then visualized using an enhanced chemiluminescence reagent (BIOMAX) and captured on an AMERSHAM imager 680 (GE HEALTHCARE).

### Immunocytochemistry

DCs were cultured on cell culture slides (SPL) for a few days, then fixed in 4% paraformaldehyde at 25 °C for 15 min before being sequentially incubated with 0.3% Triton- × 100 permeabilization solution at 25 °C for 10 min and then 5% donkey serum blocking solution at 25 °C for 1 h. Next, they were incubated with primary antibodies against LEF1 (CELL SIGNALING) and the active form of β-catenin (CELL SIGNALING) at 4 °C overnight. After washing several times with PBS, they were incubated with Alexa 594 conjugated-anti-rabbit antibodies (INVITROGEN) at 25 °C for 1 h and then counter stained with 4',6-diamidino-2-phenylindole (DAPI; INVITROGEN). Images were obtained using a Nikon Eclipse Ni-E fluorescence microscope (NIKON).

### Hair reconstitution assay

Freshly separated epidermis was incubated in PBS supplemented with 0.25% trypsin and 10 mM EDTA (GIBCO) at 37 °C for 15 min. The trypsin was then inactivated by adding FBS, and the liberated epidermal cells were then filtered through a 40 µM cell strainer (BD) before being mixed with CD1, CD3, CD5, and MS275 treated DCs. Each of these mixtures was then subcutaneously implanted into the skin on the backs of 7-week-old male BALB/c nu mice. This skin was collected 2 weeks later, and the reconstituted HFs were quantified using Image J software (NIH/FIJI).

### RNA isolation and quantitative real-time polymerase chain reaction (qRT-PCR)

We harvested total RNA from the DCs using TRIzol (INVITROGEN) and prepared complementary DNA (cDNA) using a RevertAid First Strand cDNA Synthesis Kit (THERMOSCIENTIFIC). All cDNA templates were then mixed with SYBR Green Master Mix (BIONEER), and qRT-PCR was performed on an ABI 7700HT thermal cycler. The mRNA cycle threshold (Ct) values were normalized against 36B4 for ΔCt in the same sample and then to the control sample to produce ΔΔCt. Finally, the fold change (2 − ΔΔCt ) was calculated. The primers used in this study are listed in Supplementary Table S1.

### ChIP and ChIP-qPCR analysis

First, we crosslinked both the CD3 and MS275 treated DCs in 1% formaldehyde at 25 °C for 10 min and then quenched in glycine at 37 °C for 5 min. These cells were then resuspended and lysed using an EZ-ChIP kit (MILLIPORE), and then the cross-linked DNA from the lysates were sheared using a Branson sonicator programmed as follows: 15% pulse, 0.2 s on/0.8 s off for 10 s for 7 cycles and the samples were kept on ice. The sheared DNA was then incubated with protein A/G agarose beads (SANTACRUZ) and 2 μg of the anti-acH3 antibody (MILLIPORE) overnight at 4 °C. The rest of the experiments were performed as described in the manufacturer's instructions.

Eluted ChIP-DNA samples were mixed with SYBR master mix (BIONEER) and subjected to qPCR on the ABI 7700HT thermal cycler. All qPCR reactions included primers against LEF1 and BMP4 promoter. These primer sequences are provided in Supplementary Table S2.

### ALP activity

ALP activity was evaluated using cultured DCs as follows: the cells were fixed in 4% formaldehyde at 25 °C for 10 min, rinsed twice with PBS, and then washed with ALP buffer (Tris MgCl2). Cells were stained using NBT/BCIP (ROCHE) at 37 °C in the dark according to the manufacturer’s instructions. We stopped the ALP reaction by applying ALP buffer and then rinsed the plate twice with PBS. The images were acquired using the Leica microscope system. To measuring optical density, the samples were solubilized in SDS-10% HCl buffer overnight incubation and detected at 595 nm.

### Statistical analysis

The data were analyzed using unpaired two-tailed Student’s t test and Prism 8 (GRAPHPAD, San Diego, CA) software. The data are shown as the mean ± S.E.M for three to six replicates and significance was set at *P* < 0.05.

## Supplementary Information

Supplementary Information 1.
