# Supplementary material for: Inhibition of class I HDACs preserves hair follicle inductivity in postnatal dermal cells
Source: Sci Rep. 2021 Dec 15;11:24056. doi: 10.1038/s41598-021-03508-0 (PMC8674223; doi:10.1038/s41598-021-03508-0)
Supplement: Supplementary file 1 — Supplementary Information 1. [file 41598_2021_3508_MOESM1_ESM.pdf]

Inhibition of class I HDACs preserves hair follicle inductivity in postnatal dermal cells.

Minji Park[1,2], Sunhyae Jang[1], Jin Ho Chung[1,2], Ohsang Kwon[1,2], Seong Jin Jo[1*]

Supplementary Information

Supplementary figures 1-2

Source data figures 1-3

Supplementary tables 1-2

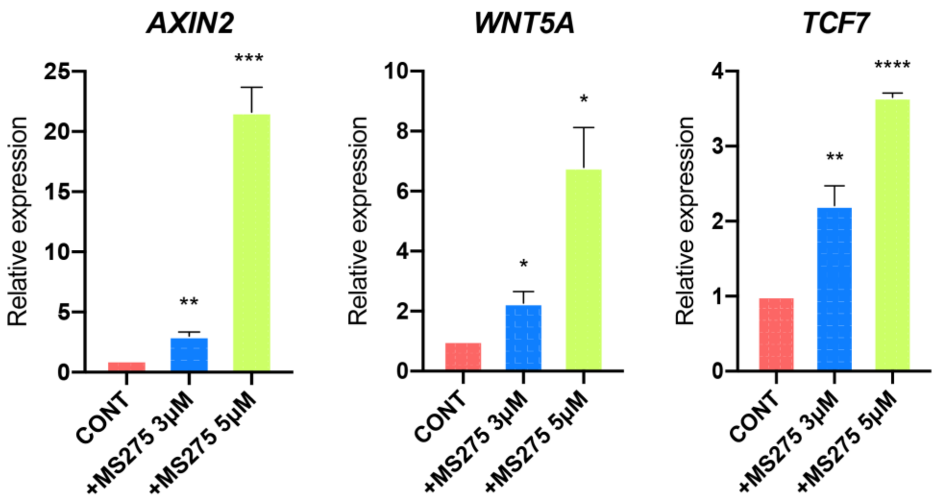

SFig1. Wnt signal pathway genes WNT5A, AXIN2, TCF7 were examined in MS275 treated DCs.

The mRNA level of AXIN2, WNT5A and TCF7 genes increased in MS275 treated DCs compared to CONT. Data are shown mean ± S.E.M. Each experiment replicate (n=3) and analyzed by t-test (*, $P<0.05$; **, $P<0.01$; ***, $P<0.001$; ****, $P<0.0001$).

CONT, controls ; DCs, dermal cells.

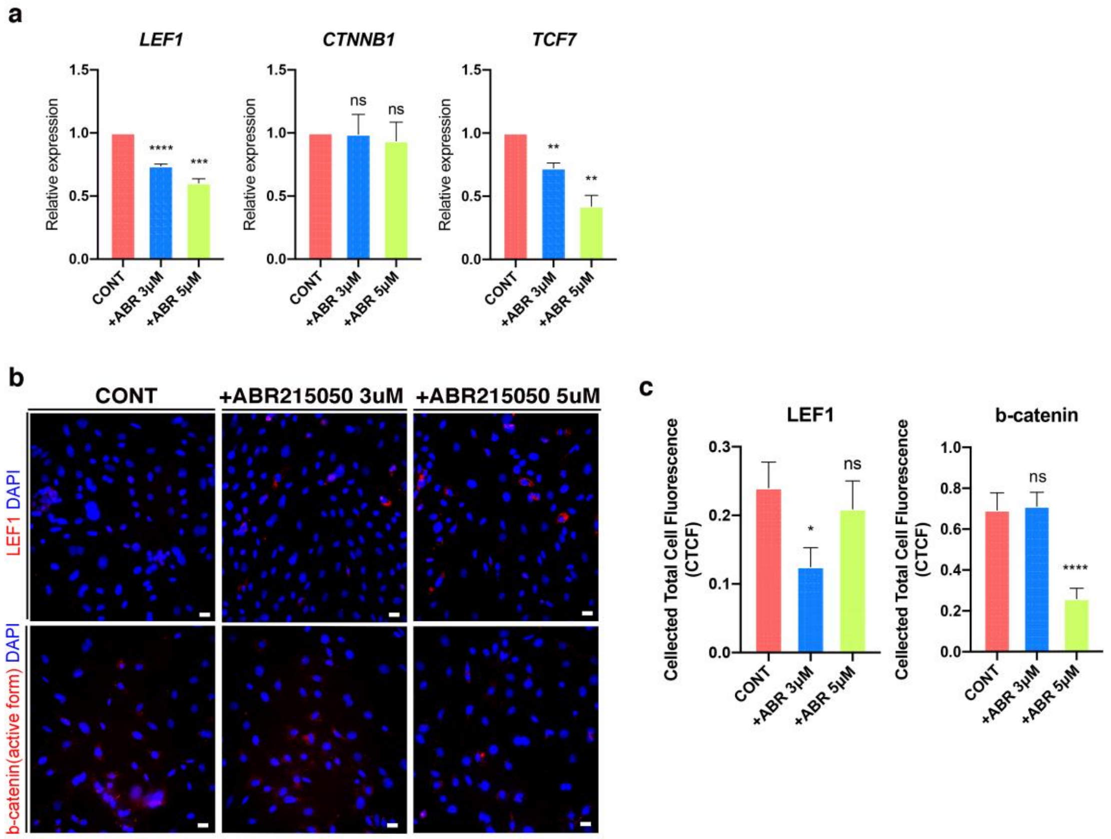

SFig2. Wnt target proteins were investigated on HDAC4 inhibitor ABR215050 treated DCs.

(a) Wnt target genes LEF1, CTNNB1 and TCF7 were not upregulated in ABR215050 treated DCs (n=3) (ns, $P\geq0.05$; **, $P<0.01$; ***, $P<0.001$; ****, $P<0.0001$). **(b)** Representative IF results were shown no significant increasing between CONT and ABR215050 treated DCs of b-catenin and LEF1. **(c)** CTCF measurement graph of IF. Data are shown as the mean ± S.E.M (n = 40) (ns, $P\geq0.05$; *, $P<0.05$; ****, $P<0.0001$).

IF, Immunofluorescence; CONT, controls; DCs, dermal cells; CTCF, collected total cell fluorescence.

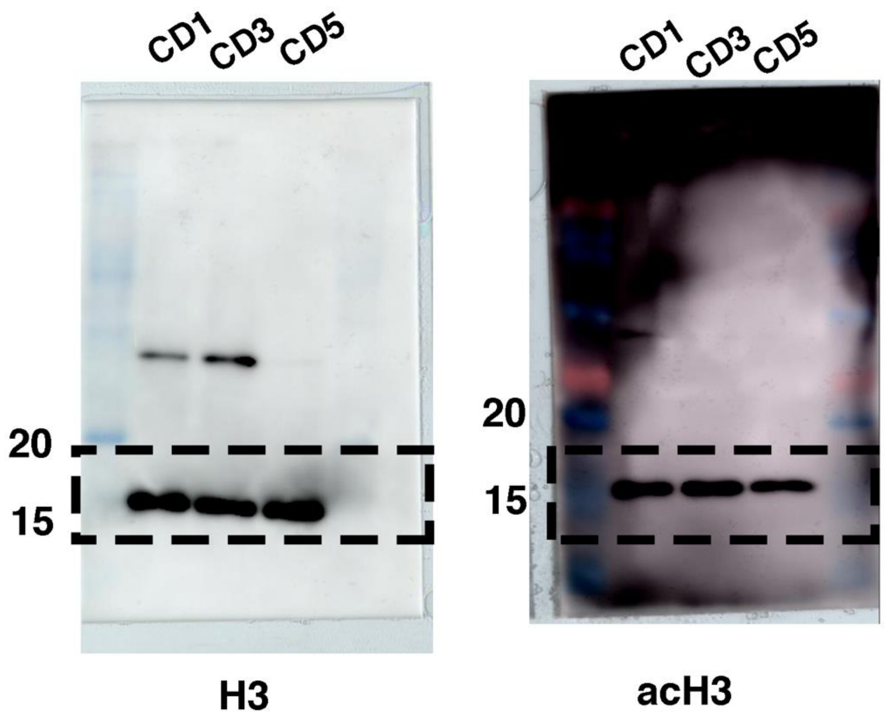

Source data figure S1. Immunoblots for acetylated histone H3 and total H3 at each day of culture.

We extracted histones from CD1, CD3 and CD5 DCs and confirmed a time-dependent decrease in histone H3 acetylation using western blot.

CD, culture day; DCs, dermal cells.

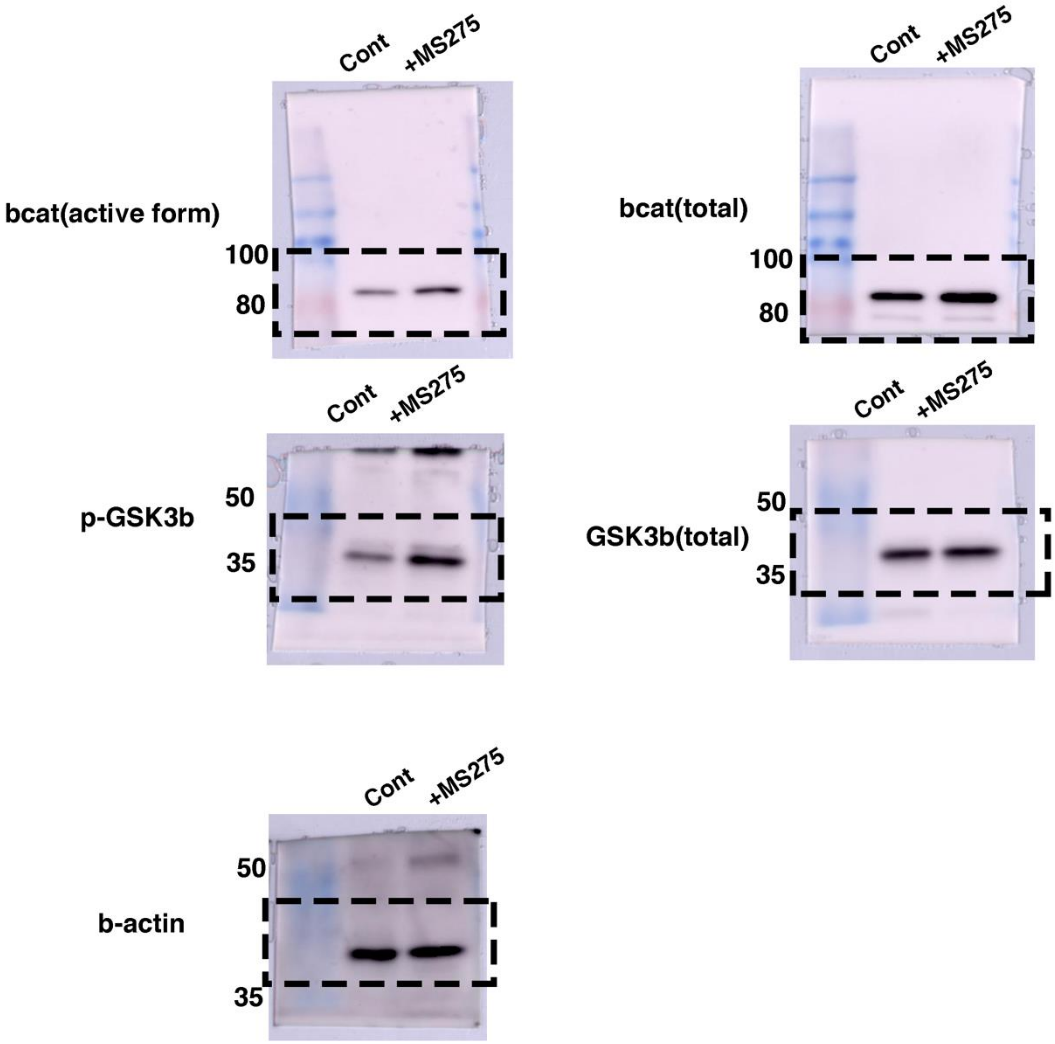

Source data figure S2. Wnt β-catenin signaling pathway molecules were examined by western blot.

To confirm Wnt-related genes expression, both nuclear and cytoplasmic proteins were extracted from control and MS275 treated DCs. MS275 activates Wnt signaling pathway through increasing GSK3β and β-catenin.

DCs, dermal cells; CONT, controls.

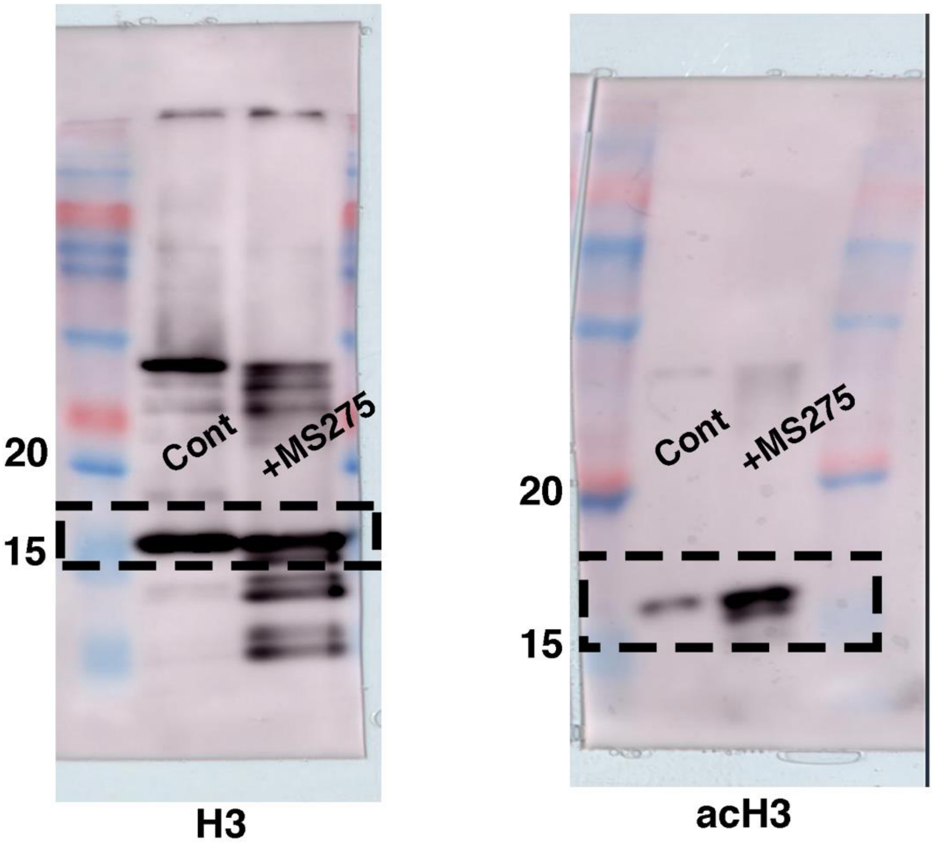

Source data figure S3. Histone H3 acetylation and total H3 expressions of MS275 treated DCs.

The western blot showed that histone H3 acetylation increased in the MS275 treated CD5

DCs.

CD, culture day; CONT, controls; DCs, dermal cells.

Supplementary Table S1. Primer sequences for real-time polymerase chain reaction.

Gene	Sense	Antisense
BMP4	ACTGAGCGCCATTTCCATGT	TAGGGAAGCACTTTGTGTGG
PROM1	TGTTCTGGTTCGGCCATAGGGAAAGCCAC	CTTGTCATAACAGGATTGTGAACACC
ALPL	CAGGTCCCACAAGCCCGCAA	CCCGGTGGTGGGCCACAAAA
HEY1	AGGTGTCTGTGCCCTGAATC	AACGGTGAAATCCGTGAGAC
CORIN	ACATCCGGTATTGCCATTTGCCTCA	CCCATAAAGTGGCCCAGTGCTT
WIF1	CAAAGAATGCCAGCCATTCC	CAGCAAAGGGACATTGACAG
LEF1	TACAACAAGGGACCCTCCTAC	GGAGAAAGGGACCCATTTGAC
CTNNB	CACAAGCAGAGTGCTGAAGGTG	GATTCCTGAGAGTCCAAAGACAG

Supplementary Table S2. Primer sequences for ChIP qPCR analysis

Gene	Sense	Antisense
LEF1	CTCGAGCCGGGAACAACAAAGA	GGGGAGAAAAAGAGAAGTTTGCC
BMP4	GCCATTCCGTAGTGCCATTC	CATGATTCTTGGGAGCCAATC
Intergenic region	TGGGCATATCCCTGGAGCTT	GGCCATCCCACAGTCACAAC
